# Utility of N-terminal (NT)-Brain Natriuretic Peptide (proBNP) in the Diagnosis and Prognosis of Pregnancy Associated Cardiovascular Conditions: A Systematic Review

**DOI:** 10.7759/cureus.32848

**Published:** 2022-12-22

**Authors:** Faith D Esbrand, Sana Zafar, Venkatesh Panthangi, Adrienne R Cyril Kurupp, Anjumol Raju, Gaurav Luthra, Mahrukh Shahbaz, Halah Almatooq, Paul Foucambert, Prachi Balani

**Affiliations:** 1 Research Department, California Institute of Behavioral Neurosciences & Psychology, Fairfield, USA; 2 Medicine Department, Osmania Medical College, Hyderabad, IND; 3 Internal Medicine, Saint Vincent Hospital, Worcester, USA

**Keywords:** high risk, peripartum cardiomyopathy, obstetrics, eclampsia, heart failure, cardiology, nt-probnp, probnp, bnp, pregnancy

## Abstract

Cardiovascular disease includes many diseases such as heart failure, cardiomyopathy, valvular disease, pericardial disease, peripheral vascular disease, rheumatic heart disease, and vascular disease to name a few. Cardiovascular disease in pregnancy is on the rise especially with women being pregnant at an older age. Brain natriuretic peptide (BNP) could be a factor in determining the severity. BNP is elevated in heart failure. This study will attempt to determine the relationship between BNP and pregnancy outcomes in women with heart failure. A keyword combination search was performed using varying databases. Inclusion and exclusion criteria were implemented and relevant articles were obtained to formulate ideas to support the topic. BNP, the amino acid peptide, is secreted by both atrial and ventricular monocytes. BNP and N-terminal (NT)-pro hormone BNP (NT-proBNP) are elevated in heart failure and seen in pregnant women alike. Within six to 12 weeks it returns to normal levels. Normal levels were shown to have good pregnancy outcomes in that the baby is healthy with normal birth weight and the mother is free of cardiovascular complications, whereas at elevated levels the pregnancy outcome was not favorable. NT-proBNP, when elevated in the pregnant patient, is a predictor of poor pregnancy outcomes, especially in patients with precursors. Testing for this peptide in pregnant women during the early stages of pregnancy could help determine the best course of action for a better outcome.

## Introduction and background

Many patients have cardiovascular illnesses as confirmed by the CDC (Center for Disease Control). It is the leading cause of mortality in pregnant women. Cardiovascular illness is growing more common in pregnant women, and it is now a primary cause of maternal morbidity. Outside of pregnancy, biomarkers such as brain natriuretic peptide (BNP) and its amino-terminal co-metabolite N-terminal pro-B-type natriuretic peptide (NT-proBNP) have been utilized to diagnose, prognosticate, and risk stratify heart failure [1,2].

In response to myocyte stretching, the 32-amino-acid peptide NT-proBNP is generated and released mostly from the ventricular myocardium. However, myocardial ischemia and infarction, as well as increased left ventricular (LV) wall stretch, cause the release of NT-proBNP. NT-proBNP levels have been associated with LV dilatation, remodeling, and dysfunction in people who have had an acute myocardial infarction. NT-proBNP is linked to body mass index (BMI), sexual hormone-binding globulin, and ethnicity [1,3]. It enhances diuresis and natriuresis while decreasing vascular tone. The physiologically inactive NT-proBNP is produced as a byproduct of BNP synthesis. The prolonged half-life of NT-proBNP, 60-90 minutes, compared to 20 minutes for BNP makes it more useful for detecting LV dysfunction. In pregnant or postpartum patients with abrupt dyspnea, obstetricians can employ BNP and NT-proBNP to detect worsening existing cardiomyopathy or new onset peripartum cardiomyopathy (PPCM). BNP levels are usually steady during a normal pregnancy, according to research [1].

Cardiovascular function and hemodynamics vary dramatically throughout the pregnancy. An increase in cardiac output (CO) is the most major change related to pregnancy. During pregnancy, CO increases due to an increase in heart rate, blood volume, and a reduction in systemic vascular resistance. Reversible cardiac remodeling includes chamber enlargement (left ventricular diastolic dimension (LVDd) and left atrial dimension (LAD), valve annular dilation, and increased LV mass). In the earlier stages of pregnancy, these changes begin and peak in the second and third trimesters (14-27 weeks and 28 weeks, respectively) [4].

PPCM is a common cause of pregnancy-related heart failure that affects women near the end of their pregnancy or soon after giving birth up to five months postpartum [3,5]. NT-proBNP levels are elevated at the time of PPCM diagnosis, according to previous studies, and a diagnosis of PPCM is unlikely if BNP or NT-proBNP levels are less than 100 pg/ml [5].

Preeclampsia is a cardiac condition that occurs during pregnancy and is caused by stress on the heart's ventricles. It is characterized by an increase in BNP levels [6-8]. The levels of BNP or NT-pro-BNP do not vary during normal pregnancy or the postpartum period, and they are only slightly raised in preeclampsia patients. On the other hand, an earlier BNP test could benefit the diagnosis of PPCM [8].

In this study, we plan to determine the relationship between how NT-proBNP can determine the diagnosis and prognosis of cardiovascular disease in pregnant patients. Our goal will be to show that NT-proBNP can assist in determining the cardiovascular health of pregnant women.

Methods

We obeyed the Preferred Reported Items for Systematic reviews and Meta-analysis (PRISMA) guidelines for conducting our systematic review [9]. We systematically searched multiple electronic databases such as PubMed and Medline for data collection. We explored databases by using keywords such as “BNP,” “pregnancy,” “heart failure,” “Preeclampsia,” and “postpartum cardiomyopathy.” The keywords were used in combination to find the relevant studies. The total number of articles found was 11,183. Table 1 shows the results of the search strategy.

**Table 1:** Keyword search results. BNP: brain natriuretic peptide PPCM: peripartum cardiomyopathy

Search Strategy	Total Number of Studies without Inclusion and Exclusion Criteria	Total Number of Studies with Inclusion and Exclusion Criteria
BNP and Pregnancy	227	22
BNP and Heart Failure	5,626	344
Heart Failure and Pregnancy	5239	270
Preeclampsia and PPCM	91	12
BNP	10	4
Total	11,193	652

Inclusion criteria

For our research, we included all the articles in English, free full text, and full text. Articles were from the last five years (2017-2022). The population was female ranging from age 13 and above. All the study subjects were human.

Exclusion criteria

Grey literature, books, documents, languages other than English, people located outside the United States, and duplicates were excluded from the study.

Results

**Figure 1:**
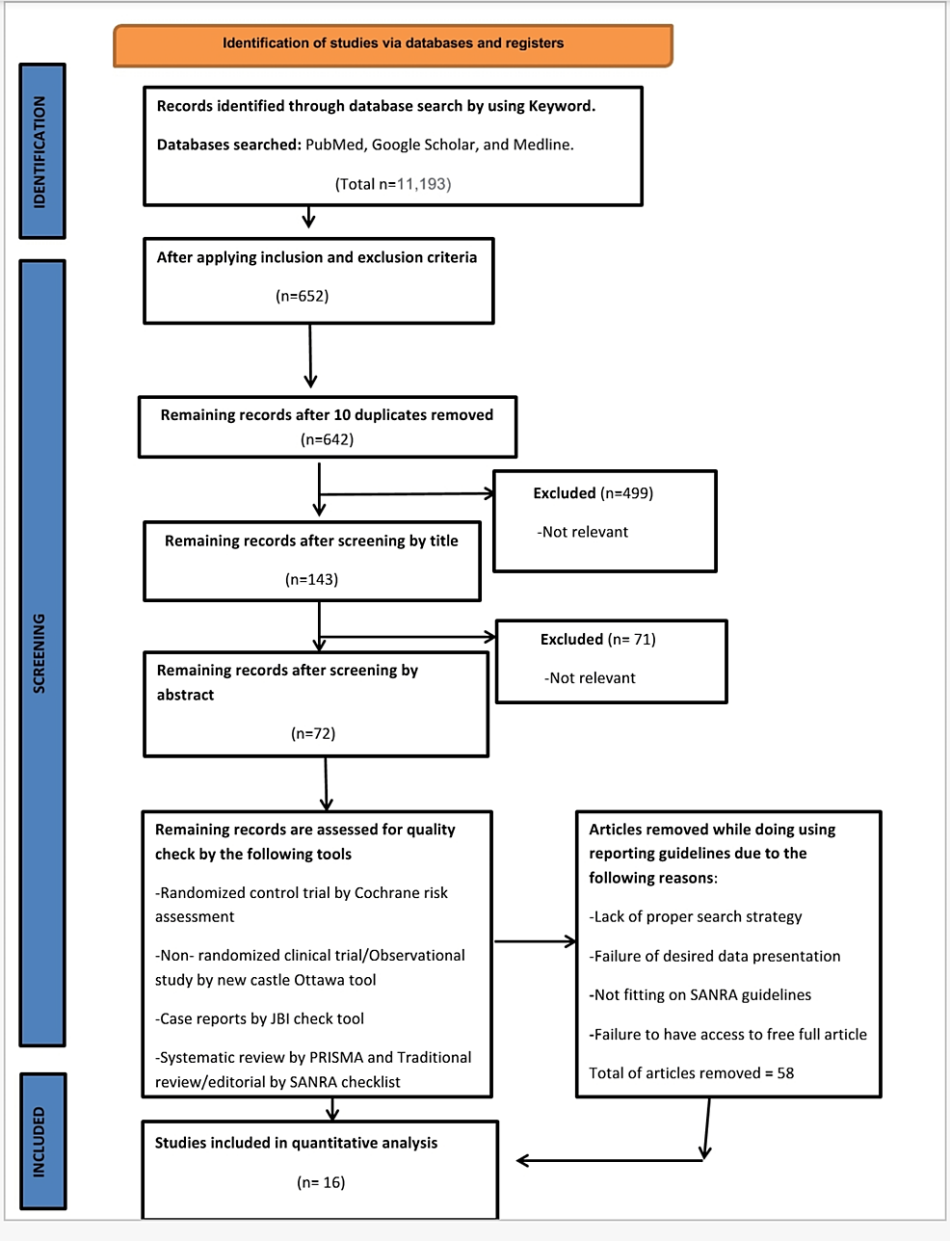
Article selection flowchart. PRISMA: Preferred Reported Items for Systematic Reviews and Meta-Analysis JBI: Joanna Briggs Institute SANRA: Scale for the Assessment of Narrative Review Articles Page MJ, McKenzie JE, Bossuyt PM, Boutron I, Hoffmann TC, Mulrow CD, et al. The PRISMA 2020 statement: an updated guideline for reporting systematic reviews. BMJ 2021;372:n71. doi: 10.1136/bmj.n71

A total of 11,193 studies were obtained from databases. Records were analyzed on the basis of title, abstract, and application of inclusion and exclusion criteria. Despite exclusion for a year, one article from 2002 was a part of the results. A total of 652 studies were obtained. After the removal of duplicates, 642 studies were left. Further, all the articles were screened on the basis of title and abstract and 72 remained. The studies were then assessed for quality, and 50 studies were included. Clinical trials were screened through Cochrane Risk Bias Assessment Tool, Observational studies by New Castle Ottawa, Systematic and Meta-analysis by PRISMA, and Literature review by Scale for the Assessment of Narrative Review Articles (SANRA) Checklist. Figure 1 shows the results of the search strategy.

## Review

Discussion

Cardiovascular disorders have been increasing in pregnant women. Heart failure in pregnancy is a very serious condition because not only is the life of the mother at risk, but also the life of the unborn child as well. BNP has been used to determine patients with heart failure the severity of the disease.

What is BNP?

The 32-amino-acid peptide hormone BNP belongs to a group of structurally related peptide hormones. They have a 17-amino-acid core loop that is connected by a disulfide bond between two cysteine residues, and carboxy- and amino-terminal tails differ from each other. Both atrial and ventricular myocytes secrete BNP, with the left ventricle being the primary source [10,11]. The BNP gene is located next to and upstream of the atrial natriuretic peptide (ANP) gene on the chromosome's distal short arm. Three exons and two introns comprise this gene. BNP's messenger RNA (mRNA) has four "AUUUAA" repeat sequences, which are thought to cause mRNA instability, so that mRNA turnover is high and BNP is produced in bursts. In some secretory granules of atrial and ventricular myocytes, mRNA-21 is translated into a 108-amino acid precursor protein (proBNP), which coexists with ANP. In hypertrophic cardiomyopathy, dilated cardiomyopathy, and heart failure, the number of these granules in normal ventricular tissue rises [10].

Where there are higher levels of NT-proBNP and BNP, they have been associated with an increased risk of death from any cause, such as cardiovascular death, sudden death, pump failure death, as well as hospitalization for heart failure [11]. Table 2 summarizes the selected studies.

**Table 2:** Gives a summary of the studies that were selected for this section. BNP: brain natriuretic peptide NT-proBNP: N-terminal pro-b-type natriuretic peptide

Study	Author	Year	Type of Study	Patients	Purpose of Study	Results	Conclusion
1	Rørth et al. [11]	2020	Randomized Control Study	8399	BNP and NT-proBNP are compared to determine the mortality and morbidity in heart failure.	Body mass index, age, and renal function all had an impact on the outcomes, however, the left ventricular ejection fraction did not. Each peptide was a reliable predictor of hospitalization for heart failure and mortality.	In patients with heart failure and reduced ejection fraction, the ratio of NT-proBNP to BNP varies between those with and without atrial fibrillation, rises markedly with age, and declines noticeably with declining renal function.
2	Cowie MR, Mendez GF [10]	2002	Review	________	Treatment for heart failure can be modified using the plasma levels of BNP.	BNP plays a role in determining heart failure in patients. The risk is elevated the higher the value of the BNP.	When diagnosing, risk-segmenting, and monitoring patients with heart failure or other cardiac dysfunction, doctors can use plasma BNP measurements to help.

Pregnancy and cardiovascular conditions associated with pregnancy

As the demographics have been changing over the years, there has also been an increase in the number of patients presenting with cardiovascular disease. In more recent years many women have delayed having children from their 20s and 30s to now during their 40s. These older-aged women present with cardiac conditions such as chronic hypertension and coronary disease [12]. In addition, the number of pregnancies among people who were born with congenital heart disease that was either corrected or is still being assessed has increased. The health of the pregnant woman with cardiovascular disease and her children is compromised since there is a higher risk of secondary illness, acute cardiovascular decompensation, premature birth, and death. Pneumovascular disease, maternal cyanosis, low maternal functional class, arrhythmias, and anticoagulant use all raise the risk of complications for both the mother and the fetus [12].

During the first trimester, peripheral vascular resistance begins to diminish due to uteroplacental shunting and a decrease in vascular sensitivity to the presser effects of angiotensin II and norepinephrine. The heart rate of the mother increases by 10 to 15 beats per minute. An increase in CO is caused by an increase in preload, a decrease in afterload, and an increase in heart rate [12]. The patient is more sensitive to difficulties at several phases of pregnancy: near the end of the first trimester, around 20 weeks gestational age in the second trimester, and around 29 to 30 weeks gestational age, when the blood volume rise is at its height. For a parturient with cardiovascular disease, however, the peripartum period is important. Due to an increase in catecholamines, the mother's heart rate and CO both increase significantly during childbirth. Each uterine contraction pumps up to 500 ml of blood into the maternal circulatory system, causing a large rise in preload. This autotransfusion lasts a long period after it is delivered [12].

PPCM has the potential to lead to long-term systolic dysfunction, particularly in black women. Pregnancy-related hypertension disorders are the primary hypertensive disorder of pregnancy (HDP) risk factor for PPCM. Additionally, PPCM often occurs earlier in women with positive HDP results than in those with negative HDP results. HDPs that raise the risk of PPCM include preeclampsia and prenatal hypertension, which are substantially more common in black women. Preeclampsia and PPCM are pathophysiologically related illnesses that are most likely triggered by the placenta's increased production of vasculotoxic and antiangiogenic hormones. Early diagnosis has been shown to have a better outcome [13]. Table 3 summarizes the selected studies.

**Table 3:** Gives a summary of the studies that were selected for this section. PPCM: peripartum cardiomyopathy HDP: hypertensive disorders in pregnancy LV: left ventricular EF: ejection fraction

Study	Author	Year	Type of Study	Patients	Purpose of Study	Results	Conclusion
1	Lewey [13]	2020	Retrospective study	220	Study the recovery of systolic function in PPCM patients by stratifying them based on HDP, the time from diagnosis, and race.	Patients diagnosed early, especially black patients, had higher rates of left ventricle recovery than those diagnosed later in pregnancy.	There is scant evidence establishing a connection between LV function recovery and early postpartum appearance and problems with pregnancy-related hypertension. The EF upon presentation is related to the diagnosis.
2	Adam [12]	2017	Review	________	To understand the physiologic cardiac changes that take place in a pregnant mother during pregnancy.	In order to have a better outcome, a multidisciplinary team is needed.	Early intervention in pregnant women with cardiac disease is best managed by understanding the physiologic changes. This allows for the determination of the mode of delivery.

Relationship of NT-proBNP in pregnant patients

BNP levels rise in patients with heart failure because ventricular stretching produced by increased filling pressure increases BNP secretion. BNP levels are typically two-fold higher during a normal pregnancy than in a non-pregnant state, and they do not alter much during pregnancy or after delivery (four to six weeks) [4]. The normal baseline for BNP in pregnant women is <100 pg/ml. If >128 mg/ml was seen around the 20-week mark of pregnancy, then the patient can be deemed to have adverse cardiovascular conditions [5]. NT-proBNP levels of 304.3 ng/L were seen in patients with preeclampsia whereas 60.8 ng/L were seen in those pregnant patients without preeclampsia [6].

NT-proBNP has a half-life of 60 to 90 minutes whereas BNP has a half-life of 20 minutes [1]. In the immediate postpartum period, both BNP and NT-proBNP are elevated and their levels return to baseline around the 6- to 12-week postpartum mark. Pregnant patients that have a known history of a cardiovascular condition have been seen to have elevated BNP and therefore need to continue care after pregnancy and they are still considered high risk [14]. Recent studies show that there is a possible relationship between NT-proBNP and pregnancy. Increased NT-proBNP levels were an independent risk factor for cardiovascular issues at 20 weeks of pregnancy in pregnant women with congenital heart disease. Normal BNP levels during pregnancy were also found to predict minimal risk in women with cardiac illness, whereas high levels were associated with poor maternal outcomes [15,16]. During this critical time, period screening for NT-proBNP during the second trimester would be appropriate to determine the risk level for both mother and baby. Table 4 summarizes the selected studies.

**Table 4:** Gives a summary of the studies that were selected for this section. BNP: brain natriuretic peptide NT-proBNP: N-terminal pro-B-type natriuretic peptide PPCM: peripartum cardiomyopathy LV: left ventricular cTnI: cardiac troponin T CHD: congenital heart defect

Study	Author	Year	Type of Study	Patients	Purpose of Study	Results	Conclusion
1	Sheikh et al. [14]	2021	Systematic Review	128	The effectiveness of BNP and NT-proBNP as diagnostic tools for cardiac disorders, like heart failure and pre-eclampsia, in expecting and recently delivered women is now under investigation.	The levels of BNP and NT-proBNP can be used to diagnose cardiac conditions such as heart failure and pre-eclampsia in women who are pregnant or have just given birth.	Cardiac disorders such as heart failure and pre-eclampsia can be identified in pregnant and recently delivered women by measuring their BNP and NT-proBNP levels.
2	Hoevelmann [5]	2021	Prospective Study	35	To determine if a high BNP level suggests that PPCM, a risk factor for heart failure in pregnant women, can impede LV recovery.	In the women who did not experience LV recovery, the baseline NT-proBNP levels were higher. NT-proBNP was less than 900 pg/ml at the time of diagnosis, according to the analysis.	NT-proBNP has been found to be prognostic in predicting LV recovery in patients with PPCM.
3	Kimura et al. [4]	2019	Cohort Study	405	The purpose of the study is to examine the relationship between the levels and changes of cardiac troponin and BNP in pregnant women.	BNP levels rose after birth and were associated with lower hemoglobin levels, enlargement of the left atrium, and an increase in the left ventricular diastolic dimension. Furthermore, cTnI levels rose to 0.015 ng/mL in 4.0% of pregnant women after delivery.	Following delivery, BNP levels increased concurrently with enlarging heart chambers and decreasing hemoglobin levels. Higher cTnI levels were also influenced by circumstances associated with labor and delivery.
4	Kumari et al. [6]	2019	Prospective Study	149	According to a high BNP level, PPCM, a risk factor for heart failure in pregnant women, can impede LV recovery.	NT-proBNP concentrations were higher in preeclampsia patients than in control patients. There were no differences between chronic and gestational hypertension.	Underlying preeclampsia is linked to elevated circulating NT-proBNP in patients with pregnancy-related hypertension. Corin, a serine protease known to convert BNP prohormone into BNP and NT-proBNP, plays no diagnostic role in preeclampsia in human pregnancies beyond natriuretic peptide levels.
5	Elkayam [15]	2018	Editorial		To determine the appropriate screening for at-risk pregnant patients with heart failure.	The scoring system to determine heart failure in pregnant women now includes patients with valves showing a high-risk score. This score should not deter women from getting pregnant.	A thorough and in-depth lesion-specific evaluation, as well as knowledge of typical cardiac physiology and hemodynamic changes during typical pregnancy, labor, delivery, and the postpartum period and their anticipated effects on the patient's cardiac condition, are necessary for an accurate risk assessment of each patient.
6	Kampman et al. [16]	2014	Observational Study	213	Identify the independent significance of NT-proBNP levels in predicting harmful cardiovascular events during pregnancy in women with CHD in addition to other parameters.	In addition to the other established indicators, NT-proBNP levels >128 pg/mL at 20 weeks of gestation were useful in predicting the incidence of serious cardiovascular events.	NT-proBNP levels >128 pg/mL at 20 weeks of pregnancy were helpful in predicting the incidence of major cardiovascular events in addition to the other recognized markers.

Limitations

This study was done in hopes of showing the necessity of NT-proBNP and its relationship to heart failure in pregnant patients. This study was limited due to not having access to physical patients to be able to perform the laboratory test to confirm. Ethnicities were not accounted for in the study. The study was limited to articles from the past five years thereby restricting data with changes relating to BNP from previous studies. Our study allows for future trials of testing to determine the severity of heart failure and to risk stratify the course of pregnancy and its outcome.

## Conclusions

The focus of the study was to show the possibility of NT-proBNP and its value in relation to pregnant patients with cardiovascular conditions. BNP has been shown to be around normal levels in pregnant patients, however, in those with underlying cardiac conditions it is elevated. Since NT-proBNP is a byproduct of BNP with a longer half-life, it can be determined that its presence would indicate that there is a correlation to heart failure in pregnant patients. Further testing would be needed to test this theory.
